# Expert Hypertension Detection System Featuring Pulse Plethysmograph Signals and Hybrid Feature Selection and Reduction Scheme

**DOI:** 10.3390/s21010247

**Published:** 2021-01-02

**Authors:** Muhammad Umar Khan, Sumair Aziz, Tallha Akram, Fatima Amjad, Khushbakht Iqtidar, Yunyoung Nam, Muhammad Attique Khan

**Affiliations:** 1 Department of Electronics Engineering, University of Engineering and Technology Taxila, Taxila 47050, Pakistan; sa.umarkhan@gmail.com (M.U.K.); fatimaamjad1997@gmail.com (F.A.); 2 Department of Electrical and Computer Engineering, COMSATS University Islamabad, Wah Campus, Wah Cantonment, Islamabad 45550, Pakistan; tallha@ciitwah.edu.pk; 3 Department of Computer and Software Engineering, College of Electrical and Mechanical Engineering, National University of Sciences and Technology, Islamabad 44000, Pakistan; khushbakht.iqtidar18@ce.ceme.edu.pk; 4 Department of Computer Science and Engineering, Soonchunhyang University, Asan 31538, Korea; 5 Department of Computer Science, HITEC University, Taxila 47080, Pakistan; attique.khan@hitecuni.edu.pk

**Keywords:** pulse plethysmograph, biomedical signal processing, feature extraction, machine learning, feature selection and reduction, empirical mode decomposition, discrete wavelet transform, hypertension

## Abstract

Hypertension is an antecedent to cardiac disorders. According to the World Health Organization (WHO), the number of people affected with hypertension will reach around 1.56 billion by 2025. Early detection of hypertension is imperative to prevent the complications caused by cardiac abnormalities. Hypertension usually possesses no apparent detectable symptoms; hence, the control rate is significantly low. Computer-aided diagnosis based on machine learning and signal analysis has recently been applied to identify biomarkers for the accurate prediction of hypertension. This research proposes a new expert hypertension detection system (EHDS) from pulse plethysmograph (PuPG) signals for the categorization of normal and hypertension. The PuPG signal data set, including rich information of cardiac activity, was acquired from healthy and hypertensive subjects. The raw PuPG signals were preprocessed through empirical mode decomposition (EMD) by decomposing a signal into its constituent components. A combination of multi-domain features was extracted from the preprocessed PuPG signal. The features exhibiting high discriminative characteristics were selected and reduced through a proposed hybrid feature selection and reduction (HFSR) scheme. Selected features were subjected to various classification methods in a comparative fashion in which the best performance of 99.4% accuracy, 99.6% sensitivity, and 99.2% specificity was achieved through weighted *k*-nearest neighbor (KNN-W). The performance of the proposed EHDS was thoroughly assessed by tenfold cross-validation. The proposed EHDS achieved better detection performance in comparison to other electrocardiogram (ECG) and photoplethysmograph (PPG)-based methods.

## 1. Introduction

Hypertension, also known as high blood pressure, is one of the most common risk factor for cardiovascular disease (CVD) [1]. It is a very common condition in which a large amount of force from the blood pushes on the walls of the arteries leading towards heart diseases [2]. The main risk factors for hypertension include age, genetics, gender, lack of physical activity, bad diet practices, high cholesterol, excessive salt consumption, less intake of vegetables and fruit, smoking, obesity, family history, and other diseases such as kidney disease or diabetes [3]. According to the World Health Organization (WHO) statistics, 1.13 million of the world population suffers from hypertension, and more men are affected than women. One out of every four men suffers from high blood pressure issues [3]. It is a silent killer that affects the most significant tissues of the human body [4]. Indeed, many people are not aware they have hypertension [5]. In the US, an estimated 13 million people are unaware of their condition [6], while in China, 59% of people with hypertension are unaware of their condition [5]. In Pakistan, 18% of the adults are affected by hypertension, and 33% of the adults above the age of 45 were affected according to the National Health Survey Pakistan [7]. Prevalence rates of hypertension based on genetic and ethnic variations ranges from about 29% for Asians, 45% for black men, and around 46.3% for women [8].

Table 1 describes a blood pressure ranges of normal and hypertension in terms of systolic and diastolic pressures. Some of the common symptoms of hypertension include headaches, dizziness, migraine, lightheadedness, changes in vision, or fainting episodes [9]. Hypertension serves as the first step towards CVDs, but the most chronic effect of unchecked hypertension is stroke, which can lead to permanent paralysis of certain body parts. Prolonged and undetected hypertension can be fatal; therefore, its detection in the preliminary stages is crucial.

Moreover, the world is currently suffering from the outbreak of a pandemic COVID-19 caused by the coronavirus SARS-CoV-2. It was reported that there are some specific comorbidities associated with a high risk of infection and increased severity of lung injury. Most of the common comorbidities in COVID-19 patients are hypertension (30%), cardiovascular disease (8%), and diabetes (19%) [10]. Therefore, it is not entirely surprising that the COVID-19 patients experiencing worst complications are hypertensive since hypertension is most frequent in older people and these elderlies are particularly at risk of being infected by a coronavirus [11]. Given the above information and statistics, it is clear that we need a technique for recognizing hypertension as early as possible to avoid significant damage to one’s body.

Various techniques including physiological signals such as electrocardiogram (ECG) and photoplethymograph (PPG) are currently being used to detect hypertension. Detection of hypertension from PPG signals (MIMIC database) using continuous wavelet transform (CWT) and the GoogLeNet deep learning model [12] achieved an F1 score of 92.55%. This work relies on a deep learning model so it requires high processing power, large scale data sets, and more training time. The authors of [13] proposed a method based on pulse arrival time (PAT) features extracted from PPG and ECG signals. The *k*-nearest neighbor (KNN) classification method was employed to predict hypertension with an F1 score of 94.84%. The research achieved acceptable results but missing consideration of preprocessing the PPG signals as PPG suffers from motion artifacts and variation in light intensity. Identification of hypertension [14] from heart rate variability (HRV) signals yielded an accuracy of 85.47% using standard deviation of all NN intervals and multiple instance learning (MIL). However, HRV feature extraction for long-term data requires significant processing resources. A model [15] to detect hypertension obtained 93.33% accuracy using Savitzky–Golay filtering (SGF), entropy features extracted from ECG, and a support vector machine (SVM) classifier. The method achieved a considerable performance on a comparatively small data set consisting of 48 participants.

In [16], the authors proposed a hypertension detection framework based on five principal components extracted from HRV signals to achieve the highest accuracy of 85.5% with quadratic discriminant analysis (QDA). Rajput et al. [17] proposed a scheme to identify the low and high risk of hypertension. The scheme yielded classification accuracy 100% using optimal orthogonal wavelet filter back (OWFB), log, and fractal dimension features extracted from ECG. Despite promising results, this work suffers from a data imbalance problem. The authors in [18] proposed a method to detect ECG hypertensive signals using empirical mode decomposition (EMD) for preprocessing of the signals, yielding an accuracy of 97.7% through the KNN classifier. The extracted features were selected physically, making this process laborious. The method was only trained on a small data set. A system to detect hypertension using morphological descriptors derived from PPG with 92.31% accuracy is discussed in [19]. Identification of hypertension patients from ballistocardiograms (BCG) is presented in [20]. The system achieved a mean accuracy of 84.4% using class association rules (CAR) classifier and morphological features. The BCG signals were collected from patients lying on a smart mattress which has a limited availability.

Medical devices in hospitals can easily get affected by electromagnetic interference (EMI) in a complex electromagnetic environment [21,22]. ECG signals are usually affected by the EMI and preconditioning circuits. Changes in temperature and ambient lighting conditions impact the PPG signal acquisition. PPG signal acquisition is exposed to motion artifacts as well [23]. The frequency of the PPG signal is about 1–3 Hz [24], so it also requires a high order filter for signal denoising. The motivation behind this research was to investigate the feasibility of a new signal modality, i.e., pulse plethysmograph (PuPG). In contrast to PPG that uses light to detect the volume of blood flow in the finger, the PuPG senses the pressure changes in blood flow.

### Main Contributions

In this research, novel PuPG signals were used to design the hypertension detection system. The PuPG signal includes considerable cardiac health characteristic information [25,26,27]. The PuPG signals are recently being used for emotions classification [28] and biometric systems [29] as well. The main contributions of this work are listed as follows:

- This is the first study that used PuPG-based signals for the detection of hypertension.
- To accurately detect the hypertension pattern, we extract a large number of multi-domain features from preprocessed PuPG signals through discrete wavelet transform (DWT) and EMD.
- To reduce the feature dimensions and redundancy while improving the discriminative power of features, we proposed a hybrid feature selection and reduction (HFSR) scheme.
- The proposed expert hypertension detection system (EHDS) comprises preprocessing through EMD, followed by the feature extraction, kernel principal component analysis (KPCA), and weighted *k*-nearest neighbor (KNN-W) classifier, achieved an accuracy of 99.4%, sensitivity of 99.6%, and specificity of 99.4%.

The rest of this paper is structured as follows: Section 2 gives details about the materials used in this study. Section 3 describes the details about the methods. Next, we present the results in Section 4 and Section 5 discuss the proposed method and its comparative analysis. Section 6 concludes this research paper.

## 2. Materials

### 2.1. Data Acquisition

In this study, a portable pulse plethysmograph (PuPG) sensor PTN-104 (NISensors, iWorx Systems Inc., Dover, UK) in combination with NI myDAQ (National Instruments Corporation, Austin, TX, USA) was used for PuPG data acquisition. PTN-104 sensor is attached to the index finger of the subject to convert pulse pressure into an electrical voltage signal. The real-time integral of its output signal produces the same volume pulse signal as the expensive infrared PPG sensor. The PTN-104 is a rugged non-magnetic accelerometer, which is made up of piezoelectric material. IX-myDAQ (National Instruments Corporation, Austin, TX, USA) is a breakout board used for connecting the PTN-104 sensor and myDAQ (National Instruments Corporation, Austin, TX, USA) for data acquisition via mini DIN7 port. NI myDAQ is a low-cost data acquisition tool that converts analog signals to digital format and allows the users to analyze real-time data in NI LabVIEW software (National Instruments Corporation, Austin, TX, USA) on PC. The sampling frequency was set to be 1 kHz for PuPG data acquisition.

**Figure 1:**
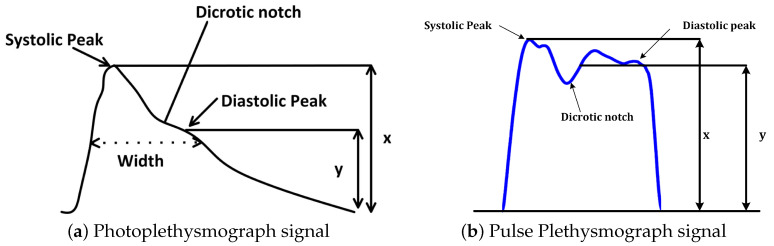
Visual comparison of Photoplethysmograph (PPG) and Pulse Plethysmograph (PuPG) signals.

It is very essential to highlight the difference between PPG and PuPG signals and sensors. Both of them operate on completely different principles with different input parameters. Table 2 presents a comparison between various properties of both sensors such as input parameters, working principles, and the impact of noise on a signal acquisition. Figure 1 illustrates the output signals acquired from both sensors. It was observed that the PuPG signal carries more information as compared to the PPG signal since multiple frequencies contribute towards the dicrotic notch for PuPG.

### 2.2. Data Set Description

**Figure 2:**
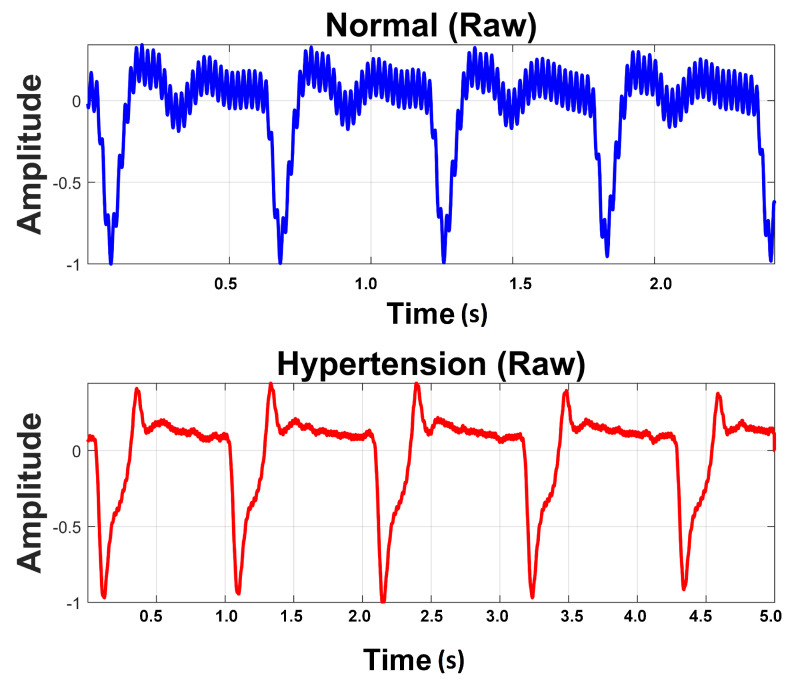
Raw PuPG signals of Normal and Hypertension classes.

Raw PuPG signals were acquired from the subjects. The data acquisition was carried out for developing a two-class data bank; one was hypertension and the other normal. A total of 700 signals were collected from hypertension subjects and 709 signals from normal subjects, with a timestamp of 10 s per signal and a sampling frequency of 1000 Hz. Subjects were advised to keep calm and remain static during data acquisition activity. Informed consent was obtained from all participants included in the research. Recording activity was performed between breakfast and lunch time. None of the involved subjects were smokers or diabetic. Table 3 shows the details of the subjects and the acquired data for this study. Figure 2 shows a comparison of raw PuPG signals collected from a normal subject and a subject suffering from hypertension. Sometimes acquired signals (both normal and hypertension) were affected by the circuit noise. The noise/power line distortion incurred due the embedded electronics of data acquisition setup can be seen as a sinusoidal oscillatory component (50 Hz) in the normal PuPG Hat of Figure 2.

## 3. Methods

### 3.1. Design of the Study

**Figure 3:**
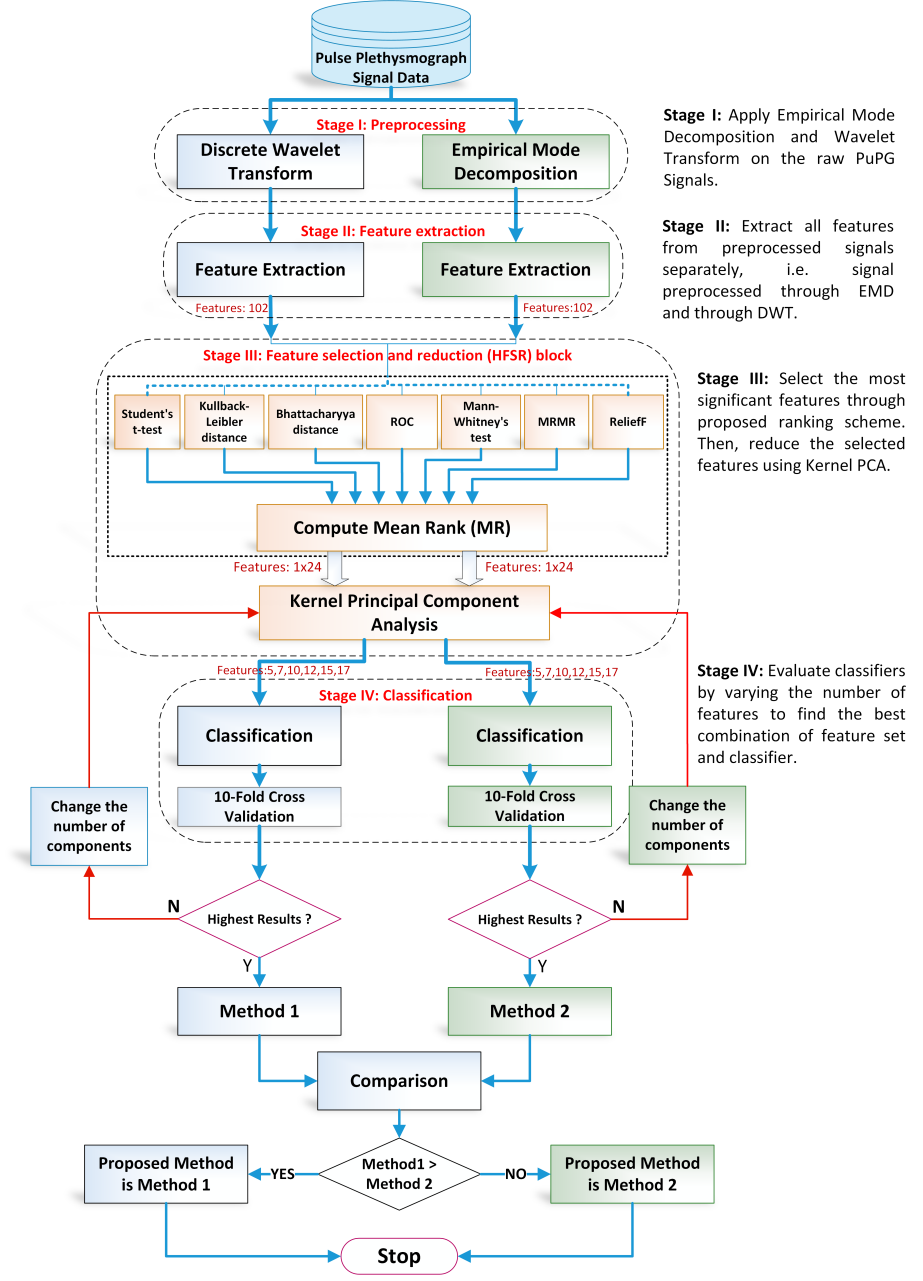
Overall flow chart of the proposed design methodology for detection of hypertension through pulse plethysmograph signals.

The proposed methodology in this research adopts the machine learning paradigm shown in Figure 3. It consists of four main stages, namely (i) preprocessing; (ii) feature extraction; (iii) hybrid feature selection and reduction, and (iv) classification. These stages are separated through a dotted line in Figure 3. Each step is elaborated in detail in forthcoming sections. This research adopts a comparative approach between two pattern analysis frameworks, i.e., method I and method II. Method I is comprised of discrete wavelet transform (DWT)-based preprocessing while method II adopts empirical mode decomposition (EMD) for signal denoising. The rest of the framework for both methods is the same. The feature values extracted, reduced feature vectors, and the performance of the classifiers vary for both methods due to the difference in preprocessing methods. All experiments were performed on MATLAB 2018a (The MathWorks, Inc., Natick, MA, USA) running on a personal computer with Core i7 (Intel Corporation, Santa Clara, CA, USA) processors and 32 GB RAM.

1. Preprocessing: It removes the irrelevant information and artifacts from the acquired PuPG signal data of normal and hypertension classes. Method I employs discrete wavelet transform (DWT) for signal denoising through frequency and mean relative energy-based criteria. Method II adopts empirical mode decomposition (EMD) for noise elimination through analysis of mean frequencies and energies of individual signal components extracted from normal and hypertension classes.
2. Feature extraction: It extracts a combination of 102 features from preprocessed PuPG through DWT and EMD separately. These include time, frequency, spectral, texture, and cepstral features. The difference between signal classes is best captured through the extraction of a wide range of informative features.
3. Feature selection and reduction: This step eliminates features with redundant information through a hybrid feature selection and reduction (HFSR) method that is a combination of multiple feature ranking and transformation schemes. A high-dimensional feature vector is reduced through a new strategy of the averaging outcome of seven feature ranking methods, thus providing more reliable results. Next, we employed kernel principal component analysis (KPCA) to further decrease the feature dimension and represent significant information in fewer parameters. Extracted features in both method I and II are fed to the HFSR scheme to reduce the dimension of the resultant feature vector.
4. Classification:The final feature vectors extracted in both methods I and II of hypertension and normal classes are fed to a range of different classifiers, i.e, support vector machines (SVM), *k*-nearest neighbors (KNN), ensemble methods, decision trees (DT), and logistic regression (LR). Classification performance of both methods is evaluated through a baseline tenfold cross-validation strategy and compared with 5-, 15-, 20-, and 25-fold cross-validation.

### 3.2. Preprocessing

The acquired PuPG data were contaminated with noise and artifacts and include redundant information (Figure 2). These noise components needs to be eliminated for a robust performance of the proposed system. Therefore, we employed DWT and EMD-based preprocessing for signal denoising. Later on, we compared the preprocessing performance of both methods.

#### 3.2.1. Discrete Wavelet Transform

**Figure 4:**
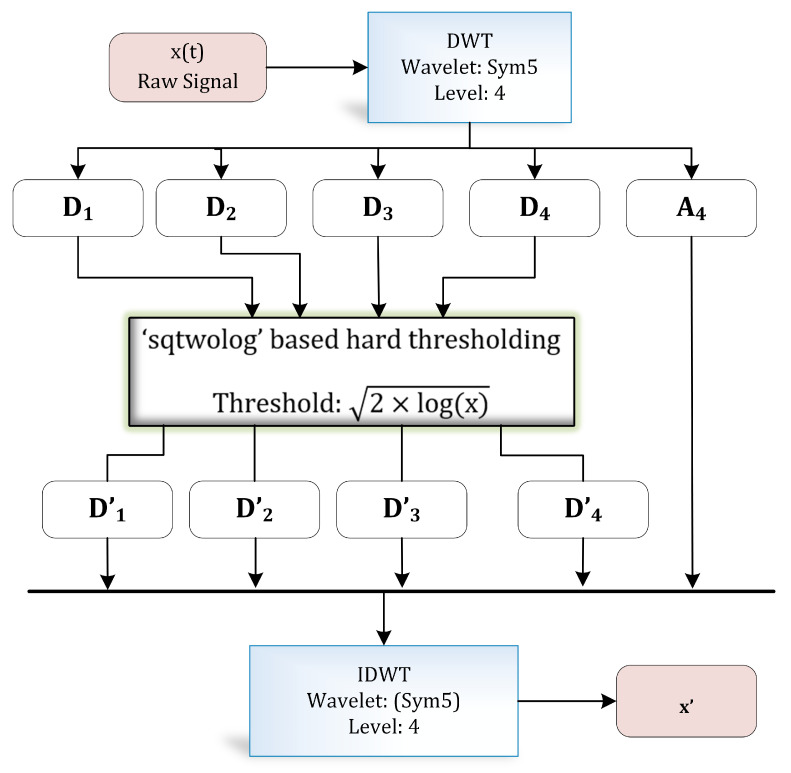
Wavelet-based denoising.

The discrete wavelet transform (DWT) is a widely applied approach in biomedical signal processing applications [30,31,32]. DWT decomposes a signal into different resolutions by using a combination of high-pass and low-pass filters. Figure 4 illustrates the complete process of wavelet-based denoising [33] adopted in this research. Numerous filter coefficients have been developed for diverse types of signal analysis applications—for instance, Daubechies, Symlets, and Coiflets coefficients, etc.

In this study, we employed the Symlet wavelet due to its similarity with the shape of the PuPG signal under consideration [34,35]. Symlet wavelet yields the best results as compared to others due to its resemblances with the morphological characteristics of the PuPG signal.

**Figure 5:**
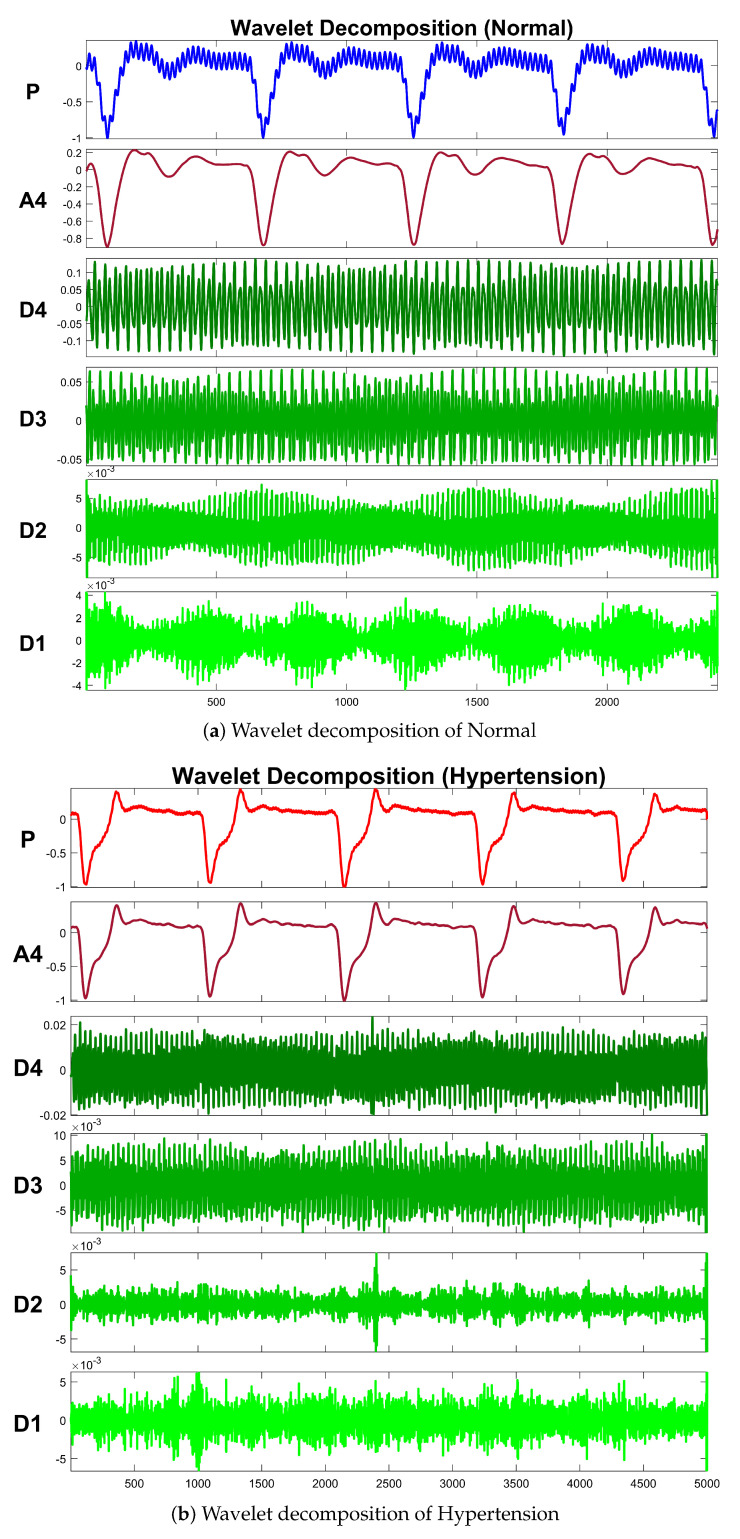
Wavelet decomposition of raw PuPG signals.

**Figure 6:**
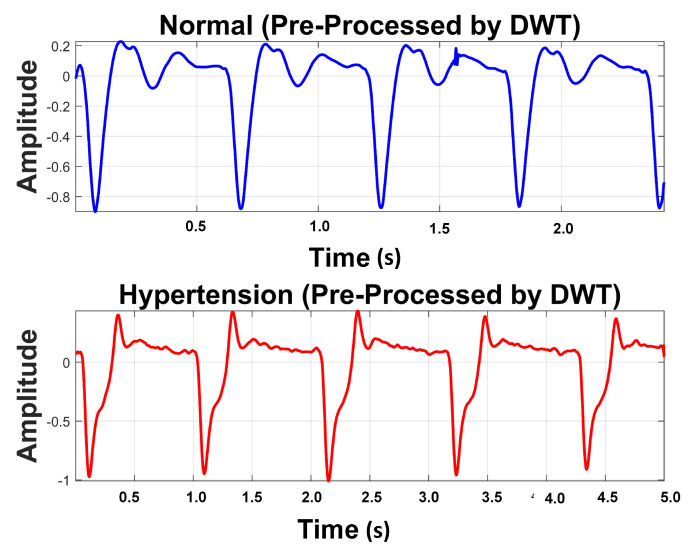
Denoised version of PuPG signal for Normal and Hypertension through DWT.

Table 4 exhibits information about decomposition levels, frequency ranges, and mean relative energies of normal and hypertension data classes of PuPG signals. It can be observed that $D_{1}$, $D_{2}$, $D_{3}$, and $D_{4}$ signal components have high frequency range and include low mean relative energies; therefore, these components were eliminated while reconstructing a denoised signal. This is also endorsed by the fact that the PuPG signal has a very low frequency (normally less than 60 Hz). Figure 5 provides a graphical illustration of wavelet decomposition for normal and hypertension PuPG signals. Figure 6 presents the denoised signal generated as a result of applying DWT. High frequency noise visible in raw PuPG signal (Figure 2) is eliminated in the denoised version.

#### 3.2.2. Empirical Mode Decomposition

EMD is an adaptive method that derives fundamental functions directly from the data [36]. EMD does not require any previously known value of the signal for its computation. The principal task for computing EMD of a given signal is to empirically determine the intrinsic oscillatory components through their particular time scales in a signal and subsequently disintegrate the signal into intrinsic mode functions (IMFs) [37]. Therefore, EMD provides remarkably better results for nonlinear and non-stationary biomedical signals.

Selection criteria of IMF have to satisfy two conditions;

- In the entire signal, the total number of local extrema and zero crossings must be equal to each other or differ by a maximum one.
- The average of the envelopes computed through local minima and local maxima must be zero.

**Figure 7:**
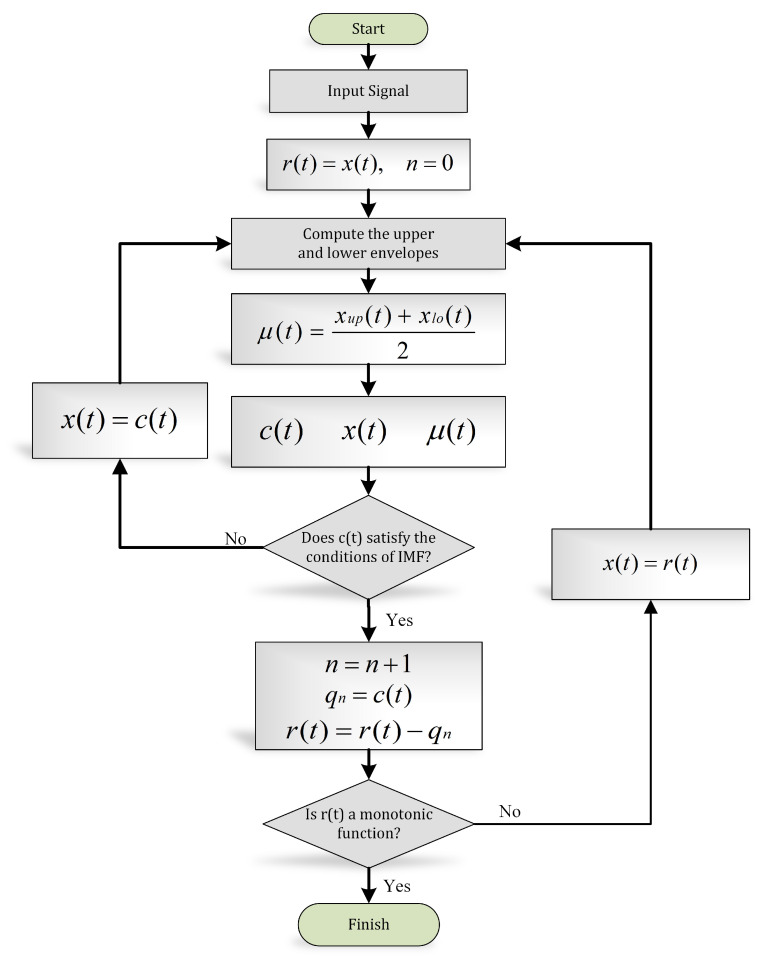
EMD algorithm (flow chart).

The systematic approach to disintegrate the signal into its IMFs is known as the “sifting” process, explained in Figure 7.

**Figure 8:**
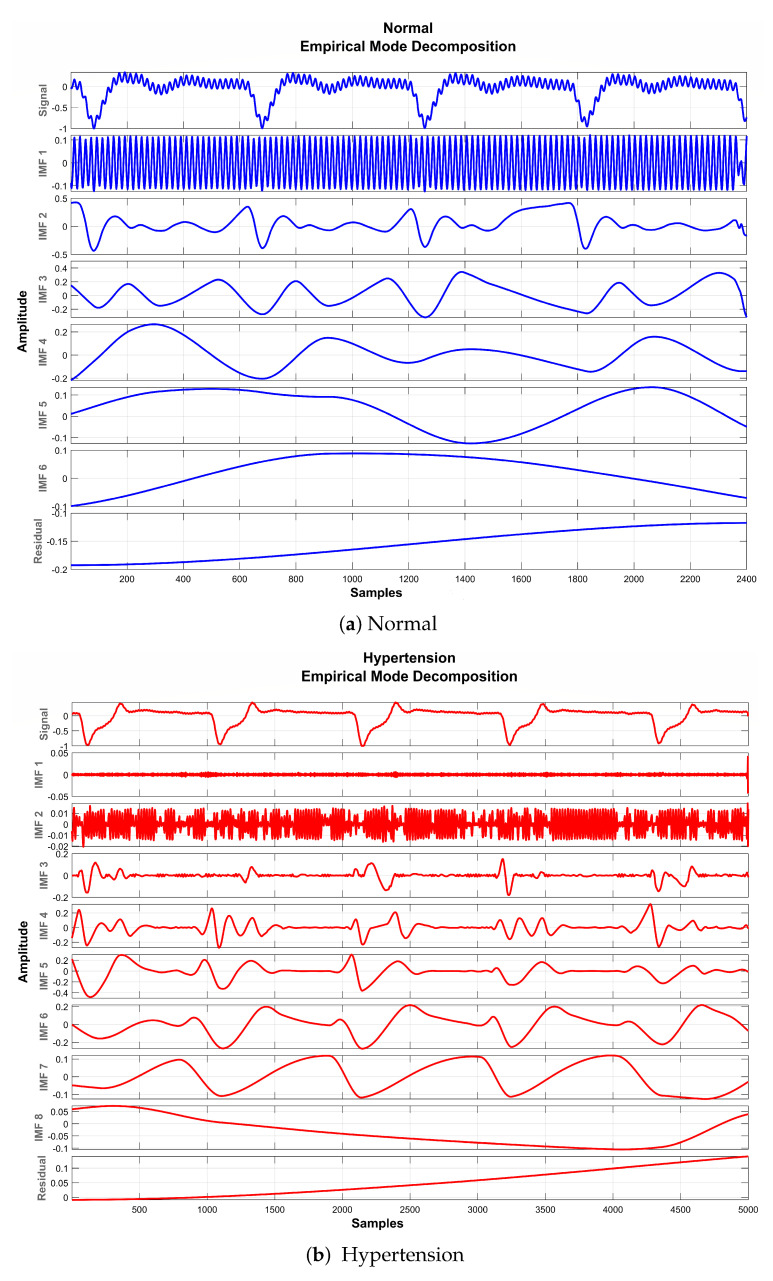
EMD decomposition of raw PuPG signals.

The basic objective of applying EMD for preprocessing the PuPG signal was to decompose the distorted signal into its constituent IMFs as depicted in Figure 8. Considering the fact that some IMFs carry discriminative and characteristic information about various data classes while others include redundant and noisy content, the determination of the proper number of IMFs is a crucial step towards creating an effective signal denoising strategy.

**Figure 9:**
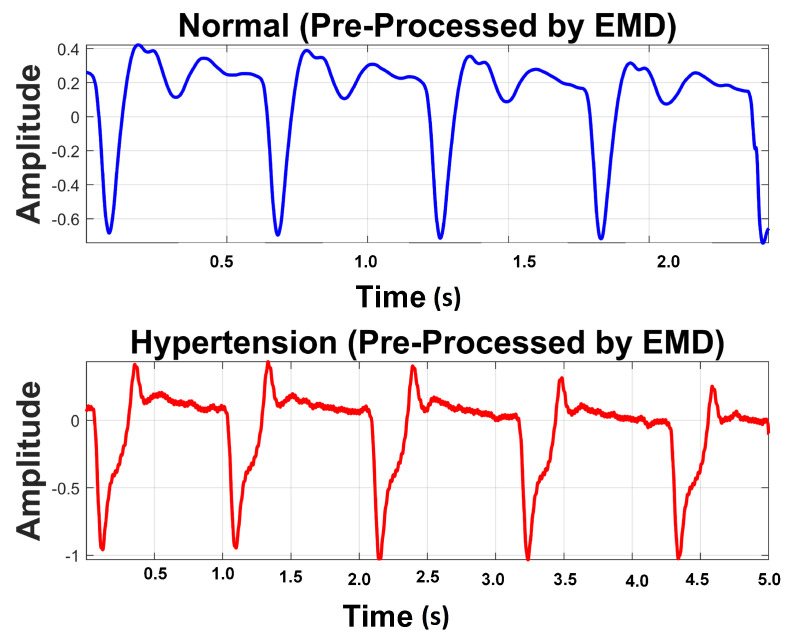
Preprocessed signal using EMD.

It is perceived from Figure 8 and Table 5 that the first IMF includes mainly high-frequency content. Table 5 provides mean frequency and energy information of each IMF for normal and hypertension data classes of the PuPG signal. The first IMF also holds very little mean relative energy components for both classes, i.e., 0.00% and 1.02% for normal and hypertension classes, respectively. Therefore, it was discarded while reconstructing the denoised signal. All other IMFs and residual signals were added to form a denoised version of the PuPG signal. Figure 9 illustrates the PuPG signal denoised through the EMD process for normal and hypertension data. It is clear that high frequency noise that was visible in raw PuPG signal (Figure 2) is eliminated now.

### 3.3. Feature Extraction

The objective of the feature extraction stage is to extract significant features from the biomedical signals of various classes that contributes towards an effective classification performance. In this study, a total of 102 features were separately computed from the PuPG signal denoised through DWT and EMD. Table 6 lists all the extracted features along with their statistical measures of mean and standard deviation (STD) for method I (DWT) and method II (EMD). We extracted time domain [38,39,40,41,42,43,44,45], spectral [46,47], fractal and chaos [48,49], chroma [50,51], cepstral [52], and texture features [53] and analyzed them statistically.

These features were subjected to the feature selection step (HFSR) to recognize the features with maximum discriminative content among normal and hypertension classes.

### 3.4. Hybrid Feature Selection and Reduction

**Figure 10:**
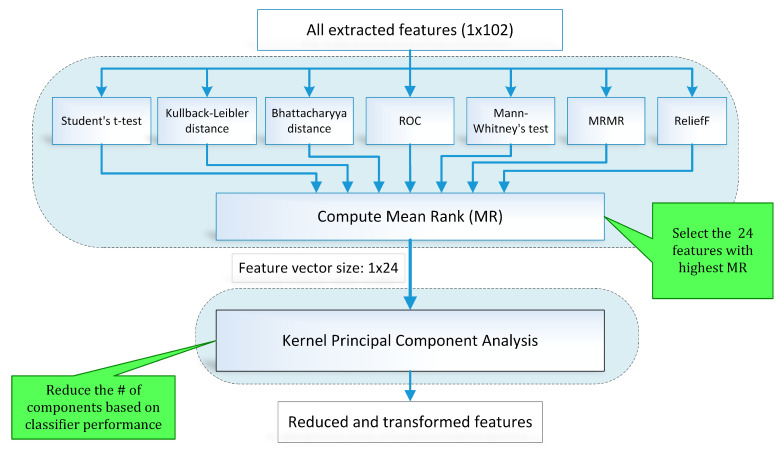
Block diagram (feature selection and reduction method).

Feature selection is one of the key steps in the modern pattern recognition and machine learning paradigms. The extracted features may include redundant information and irrelevant and noisy parameters. A two-stage hybrid feature selection and reduction (HFSR) strategy was designed to select and transform the best distinctive features as shown in Figure 10. The first stage ranks the input features through seven different methods and the second stage transforms the selected ranked features to further reduce dimensionality.

#### 3.4.1. Feature Selection Scheme

Feature selection routines serve to enhance the performance of classifiers by reducing the feature dimensions as well as decreasing the computational time [54,55]. Feature selection methods are categorized as filter methods and wrapper methods. Filter type feature selection methods employ feature ranking techniques based on the applied statistical measure for selecting a suitable feature. In wrapper type feature selection techniques, a feature subset is selected recursively based on the overall model performance. The selection criterion computes the variation in model performance that decides the addition or removal of a feature from the subset.

To address the limitations of individual feature selection approaches, we employed a hybrid scheme of feature selection by combining seven feature ranking methods through a voting strategy. Figure 10 illustrates the hybrid scheme of feature selection and reduction. In this scheme, seven state-of-the-art feature ranking techniques, namely student *t*-test (TT), Kullback–Leibler distance (KLD) [56], Bhattacharya distance (BD) [57], Mann–Whitney’s test (MWT), ReliefF (RRF) [58], minimum redundancy maximum relevance (MRMR) [59,60], and receiver operating characteristic curve (ROC) were employed to rank the feature individually. Ranking assigned to each feature by all feature ranking methods is combined to calculate the mean rank (MR) value. A threshold is applied to MR value for feature selection.

Table 7 provides the sorted lists of the best forty features with the highest MR values for method I (features extracted from signal preprocessed through DWT). Rank assigned to individual features by each ranking method is also computed. The top 24 features are highlighted in Table 6 were forwarded to the next stage. It was perceived that if a feature ranking method assigns a high rank to a particular feature that failed to get high scores from other methods, it gets rejected due to the hybrid scheme of feature selection. For instance, consider the Root Sum of Squares feature that received the rank value of 99 from the ROC method, but gets scores of 53, 49, 52, 18, 58, and 72 from TT, KLD, BD, MWT, MRMR, and RRF, respectively. It achieved an MR value of 57.29 that is below the selection criterion, so it was rejected from the final feature vector of 1 × 24 dimensions. Table 8 enlists the top forty features with the highest MR values for method II, i.e., features extracted from the signal preprocessed through EMD. The rank value assigned by an individual feature ranking method to a specific feature can be examined. One to one comparison of the top ten MR values of method I in Table 7 and method II in Table 8 reveals that the magnitude of MR values of method II (81–70) is higher than that for method I (73–67).

#### 3.4.2. Feature Reduction Using Kernel PCA

PCA applies orthogonal transformation to transform a group of likely correlated features into a set of linearly independent features known as principal components. These principal components represent the normalized linear combinations of the original features. It includes information about the most powerful variations present in the data set. The first principal component holds maximum variance information of the data set.

Kernel PCA (KPCA) [61,62] enhances the original PCA to non-linear data distribution problems through a kernel function. A kernel function projects low-dimensional feature data to a higher-dimensional feature space, where it becomes linearly separable [63].

The previous stage of hybrid feature selection reduced the feature dimensions to 1 × 24 which are fed to KPCA to further decrease dimensions for both methods I and II. Components of KPCA were selected recursively based on the classification performance through tenfold cross-validation. Separate sets of 5, 7, 10, 12, 15, and 17 components were picked for methods I and II to investigate the classification performance for differentiating normal and hypertension signal classes of PuPG signals.

### 3.5. Classification

To perform the classification of normal and hypertension classes of PuPG signal data set, this study employed a range of classification methods through tenfold cross-validation schemes. The classification methods opted in this study were SVM-Linear (SVM-L), SVM-Quadratic (SVM-Q), SVM-Cubic (SVM-C), SVM-Fine Gaussian (SVM-FG), SVM-Medium Gaussian (SVM-MG), KNN-Fine (KNN-F), KNN-Medium (KNN-M), KNN-Cosine distance (KNN-Cos), KNN-Cubic (KNN-C), KNN-Weighted (KNN-W), Decision Trees (DT), Linear Discriminant (LD), Logistic Regression (LR), Gaussian Naive Baise (NBG), Kernel Naive Baise (NBK), Ensemble Boosted Trees (Eboost), Ensemble Bagged Trees (EBT), Ensemble Subspace Discriminant (ESD), and Ensemble Subspace KNN (ESKNN). The tenfold cross-validation was also compared with 5-, 15-, and 20-fold cross-validation and 80–20% and 75–25% train-test experiments. All experiments were implemented on MATLAB 2018a on a personal computer with Core i7 with 32 GB RAM.

## 4. Results

In this study, the PuPG signal data set comprising two classes (Normal and Hypertension) was first preprocessed through DWT and EMD to develop methods I and II respectively. We obtained 102 features for each method, i.e., DWT and EMD. These features were subjected to the HFSR framework to reduce the computational complexity and feature vector dimensions. Standard statistical parameters of Accuracy (Acc), Sensitivity (Sen), Specificity (Sp), and Error rate (Err) were used to measure the classification performance.

### 4.1. Method I

In this research, a comparative analysis was performed via preprocessing the PuPG signal through DWT and EMD. This section presents the results yielded by preprocessing through DWT and succeeding processes of feature extraction, selection, and classification. Various feature sets, namely $S_{1}$, $S_{2}$, $S_{3}$, $S_{4}$, $S_{5}$, and $S_{6}$ were formed by randomly choosing 5, 7, 10, 12, 15, and 17 transformed features. These feature components were fed to several classification methods to examine the diagnostic performance through tenfold cross-validation. Table 9 presents consolidated result analysis of various classification methods for features sets $S_{1}$ (5 components), $S_{2}$ (7 components), and $S_{3}$ (10 components). Table 10 illustrates comprehensive analysis of classification performance over different classifiers for feature sets $S_{4}$ (12 components), $S_{5}$ (15 components), and $S_{6}$ (17 components). As expressed in Table 10, Ensemble Subspace KNN classifier scores highest average accuracy of 98.4%, for 12 feature components, i.e., $S_{4}$ feature set.

**Figure 11:**
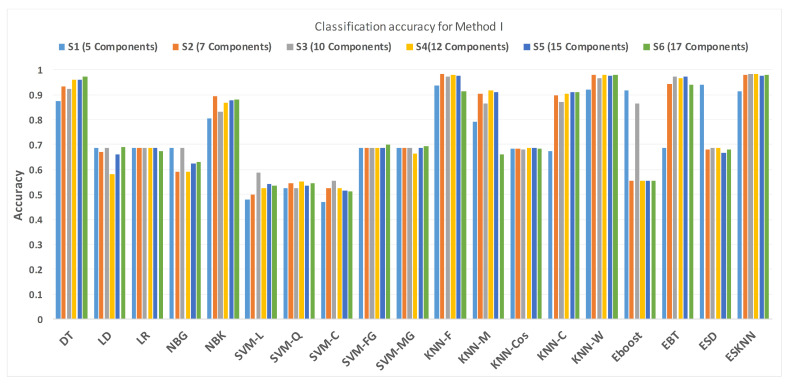
Performance of accuracy for different feature sets in various classifiers for PuPG signal through method I.

**Figure 12:**
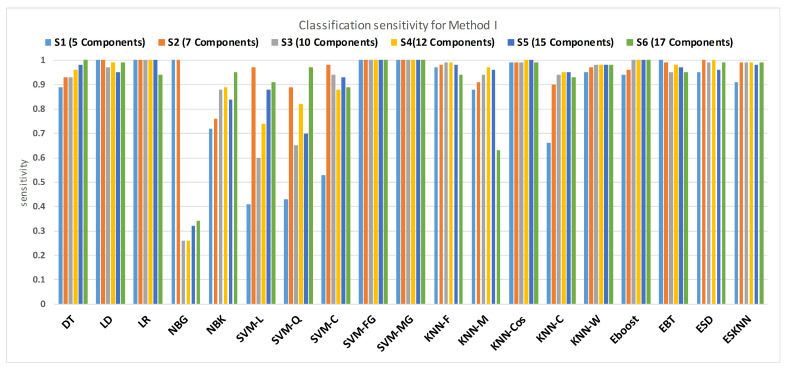
Performance of sensitivity for different feature sets in various classifiers for PuPG signal through method I.

**Figure 13:**
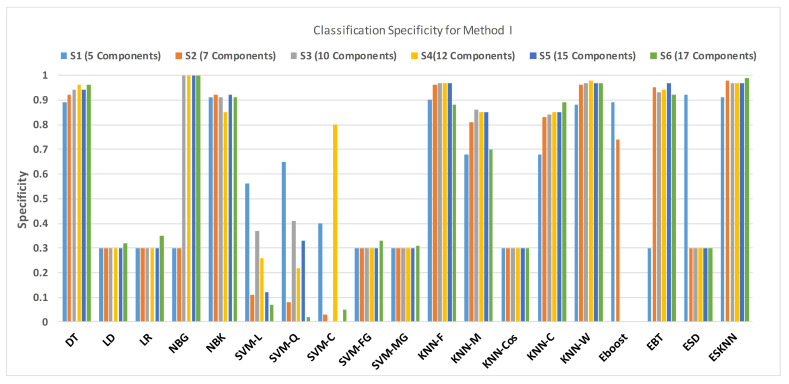
Performance of specificity for different feature sets in various classifiers for PuPG signal through method I.

Figure 11 shows the performance in terms of accuracy for different feature sets in various classifiers for distinguishing normal and hypertension classes using PuPG signals. Figure 12 demonstrates the specificity performance of several classifiers for various features sets from DWT based preprocessing method. Figure 13 presents a graphical comparison of the sensitivity performance of several classifiers for different feature combinations.

NBG classifier achieves highest specificity performance of 100% for feature sets $S_{3}$, $S_{4}$, $S_{5}$, and $S_{6}$ (Figure 12), but it reaches maximum sensitivities of 26%, 26%, 32%, and 34% for the same feature sets (Figure 13); therefore, it results in significant reduction of overall classifier accuracy of NBG. The sensitivity performance is 100% for several classifiers (LD, LR, NBG, SVM-FG, SVM-MG, EBT) for feature set $S_{1}$ (Figure 13), but the specificity performance is comparatively low.

**Figure 14:**
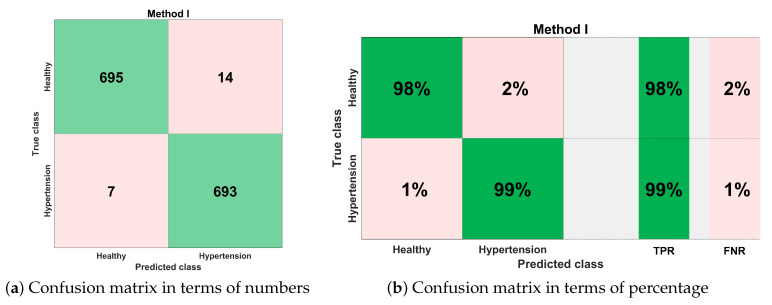
Confusion matrix for method I.

Figure 14 shows the classification performance results in the form of a confusion matrix for best configurations such as ESKNN classifier with $S_{4}$ (12 feature components). The sensitivity of classification is 99%, which means that out of 700 PuPG signals of hypertension, 693 were correctly predicted as hypertension data class while testing, whereas only seven were misclassified as healthy class. The classifier achieved a 98% specificity performance. Out of 709 healthy PuPG signal samples, 695 were accurately predicted as healthy class, whereas the remaining 14 signals were misclassified.

Table 11 includes the extensive experimentation results to avoid the classifier overfitting. The selected configuration was tested through 5-, 10-, 15-, and 20-fold cross-validation and 20% and 25% train-test holdout validations.

### 4.2. Method II

This section is primarily focused on the second method that is under discussion for this research. It encompasses the results of the classification of the features extracted after the preprocessing of the PuPG signal via EMD. A certain number of feature sets were chosen that were the result of the HFSR. The feature sets comprising of 5, 7, 10, 12, 15, and 17 transformed features were chosen and named $S_{1}$, $S_{2}$, $S_{3}$, $S_{4}$, $S_{5}$, and $S_{6}$, respectively. These feature components were fed to a various number of classifiers for classification and their performance was tested through tenfold cross-validation.

Table 12 depicts the results obtained after the classification of the feature set $S_{1}$ (5 components), $S_{2}$ (7 components), and $S_{3}$ (10 components) on using a selection of various classifiers. Table 13 shows the outcomes of various classification techniques applied on feature sets $S_{4}$ (12 components), $S_{5}$ (15 components), and $S_{6}$ (17 components). Analysis of both Table 12 and Table 13 show that a maximum average accuracy using the least number of features is 99.4%. This accuracy is the result of the weighted KNN classification method applied on the feature set $S_{1}$.

**Figure 15:**
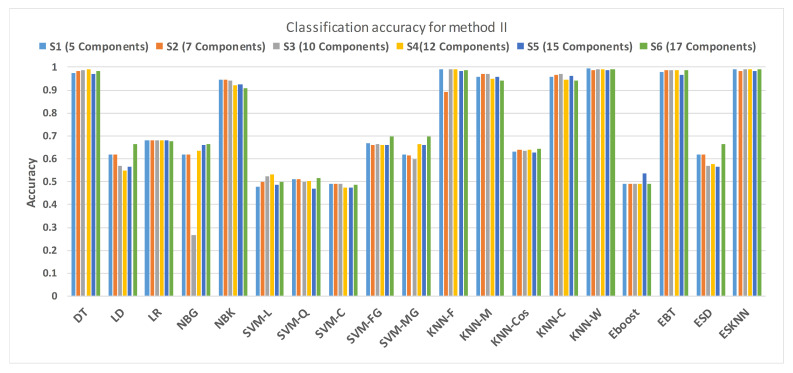
Performance of accuracy for different feature sets in various classifiers for PuPG signal through method II.

**Figure 16:**
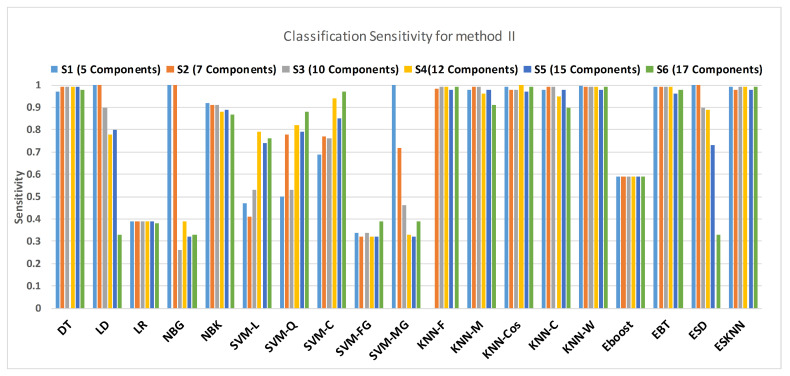
Performance of sensitivity for different feature sets in various classifiers for PuPG signal through method II.

**Figure 17:**
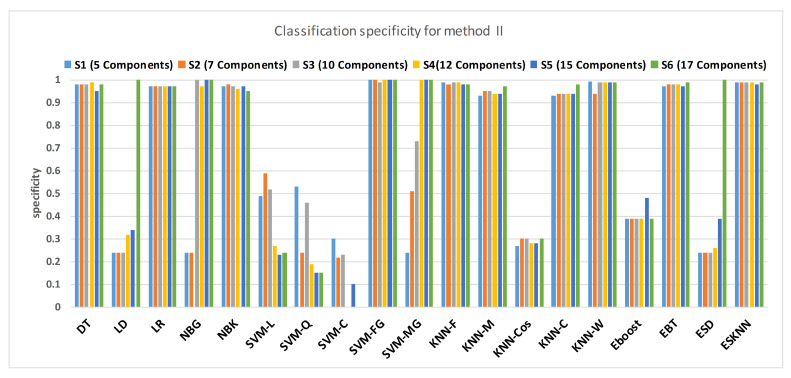
Performance of specificity for different feature sets in various classifiers for PuPG signal through method II.

Figure 15 shows a comparison of the performance of various classifiers based on the accuracy achieved as a result of distinguishing hypertension and normal PuPG signal. Figure 16 depicts the comparison result of various classifiers based on their specificities after using EMD as the preprocessing technique. Figure 17 represents the comparison of the sensitivities of various classification methods. NBG classifier achieves the highest specificity performance of 100% for feature sets $S_{3}$ (Figure 16), but it reaches maximum sensitivities of 26% for the same feature set (Figure 17). The sensitivity performance is 100% for several classifiers (LD, NBG, SVM-MG, ESD) for feature set $S_{1}$ (Figure 17), but the specificity performance is comparatively low.

**Figure 18:**
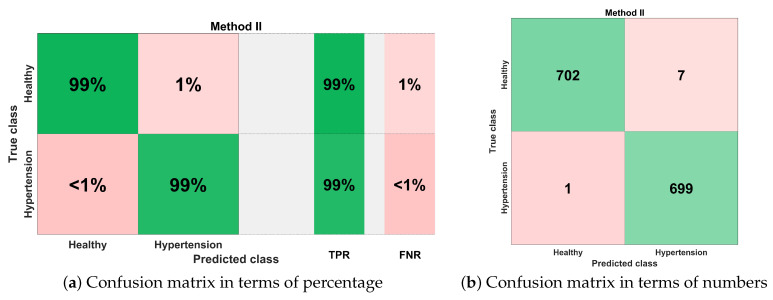
Confusion matrix for method II.

Figure 18 illustrates the best classification performance in the form of a confusion matrix for selected features set ($S_{1}$) with KNN-W classifier. The sensitivity of classification is more than 99%, which means only one out of 700 PuPG signals was wrong predicted as hypertension data class, whereas the remaining 699 PuPG signals were correctly identified as hypertension. Out of 709 healthy PuPG signals, 702 were correctly predicted as healthy, achieving specificity of 99%. The overall average classification accuracy in the best configuration with the KNN-W classifier was 99.4%.

Table 14 includes the results of comprehensive experimentation which is performed to avoid the classifier overfitting. The selected framework was examined through 5-, 10-, 15-, and 20-fold cross-validation and 20% and 25% train-test holdout validations. For all experimental settings, the proposed scheme achieved more than 98% accuracy.

### 4.3. Method I versus Method II: A Comparative Analysis

This section aims to compare both methods I and II analytically. Based on this comparison, we figure out the best working solution for the detection of hypertension through PuPG signals. Method I comprises of preprocessing of PuPG signals through DWT, followed by feature extraction. Extracted features were subjected to the HFSR scheme and finally classified through Ensemble Subspace KNN. Method II consists of EMD-based signal preprocessing followed by feature extraction. Features were fed to KNN-W classifier for distinguishing normal and hypertension data classes after being reduced through the HFSR approach.

Table 15 shows the performance comparison of methods I and II in terms of average accuracy, sensitivity, specificity, error, and number of features. Method I achieves classification performance of 98.4% accuracy, 97% sensitivity, and 99% specificity using 12 transformed features. Method II obtains 99.4%, 99.2%, and 99.6% results of classification accuracy, sensitivity, and specificity respectively through only five reduced features.

Comparative analysis of both methods establishes that method II outperforms method I in terms of achieving better classification accuracy on a reduced number of features. This might be due to the fact that the accuracy achieved in the case of DWT highly depends on the proper wavelet basis selection [64]. The selection of an appropriate basis is challenging especially for non-stationary data [65]. On the other hand, EMD is a fully data-driven, adaptive, and basis-less transformation [66]. Moreover, the IMF selection process of EMD based on relative energy and mean frequency has assisted the selection of useful discriminative signal characteristics.

**Figure 19:**
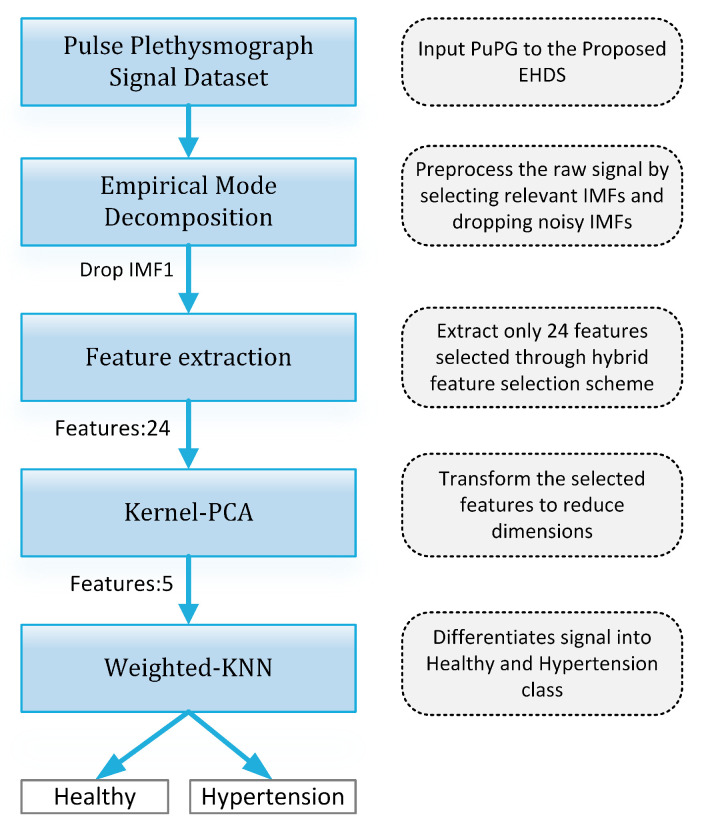
Proposed EHDS block diagram.

**Figure 20:**
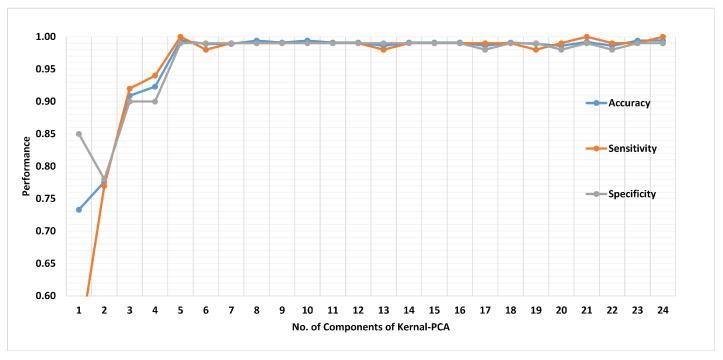
Performance of method II in terms of accuracy, sensitivity, and specificity for 1 to 24 transformed features.

Figure 19 presents the finalized EHDS (expert hypertension detection system) based on PuPG signal analysis. EHDS first takes raw PuPG signal as input and performs preprocessing through EMD by rejecting the irrelevant IMFs. Next, only 24 significant features highlighted by the hybrid selection scheme are extracted and reduced through KPCA. The final transformed 1 × 5 feature vector is fed to KNN-W to distinguish the normal and hypertension data classes. Figure 20 illustrates the classification performance of the proposed EHDS as a function of the number of transformed features. It can be observed that the proposed EHDS achieves the optimum performance on only five transformed features. The classification performance shows no notable improvement with the increase in the number of features.

## 5. Discussion

Human blood vessels and the microcirculation system experience transformations with the rise in blood pressure (BP); these changes are exceptionally obvious for patients with severe hypertension. PuPG signals carry a wealth of information about the cardiac health [25,26,27]. The PuPG signal reflects physical changes in blood volume pressure in blood vessels during the cardiac cycle. The features extracted in this study indicate the changes in Normal and Hypertension PuPG signals acquired from various subjects. The high classification performance of EHDS reflects the association of extracted transformed features with the physiological characteristics of the cardiac condition of the subject. Thus, the proposed expert system may provide a good approximation of the presence or absence of non-communicable diseases such as hypertension.

Table 16 presents a performance comparison of the recent studies. A diagnostic index for the classification of low and high-risk hypertension classes attaining accuracy of 100% was proposed by [17]. In contrast, our work is targeted towards the classification of Normal and Hypertension classes through PuPG signals. In another study, [18] developed a computational intelligence tool based on ECG signals for the classification of normal and hypertension. EMD was employed in the signal preprocessing stage, followed by nonlinear feature extraction from the decomposed IMFs. Extracted features were ranked through Student’s *t*-test. The highest classification accuracy of 97.70% was obtained through the KNN classifier with tenfold cross-validation. A photoplethysmograph (PPG) based detection of hypertension was proposed by [19]. A total of 125 features of various types were extracted and reduced through MRMR. The authors reported the best classification performance with KNN-W, specifically to be 100%, 85.71%, and 92.31% for positive predictive value, sensitivity, and F1-score, respectively.

The current research is focused on the classification between normal and hypertension data through PuPG signals. To the best of author’s knowledge, this is the first study that uses the PuPG signals for discriminating among normal and hypertension with high precision. The current method achieves better performance than the existing ECG- [15,17,18], PPG- [12,19], HRV- [14,16], and BCG-based [20] approaches. Our method also outperforms the fusion-based method for detection of hypertension that utlized a combination of PPG and ECG [13].

The proposed expert system could play a vital role in the early detection of hypertension in low- and middle-income countries. It is important to mention that an estimated 1.04 billion population suffered from hypertension in 2010 [67]. A non-invasive technique based on PuPG signals analysis proposed in this research could be used for the detection of non-communicable diseases.

## 6. Conclusions

Early detection of hypertension or high blood pressure is extremely significant since it does not cause any obvious symptoms in many people; hence, it can harm the heart, the kidneys, and even the brain. In this study, we proposed an automated detection system for hypertension from PuPG signals for timely and precise screening of disease. First, PuPG signals were preprocessed through EMD, followed by feature extraction of various types. Highly discriminative features were selected through the proposed HFSR scheme that consisted of feature reduction and selection methods. The resultant reduced features of dimension 1 × 5 were subjected to various classification methods. The KNN-W classifier achieved the best performance in terms of accuracy, sensitivity, and specificity of 99.4%, 99.2%, and 99.6%, respectively. To compute the model performance and avoid overfitting, 5-, 10-, 15-, and 20-fold cross-validations were employed. The proposed method was also compared with the DWT based preprocessing scheme followed by the same feature extraction, selection (HFSR), and classification pipeline. The main advantages of this research are as follows:

- The proposed EHDS system is based on the non-invasive methodology of PuPG signals.
- The EHDS is reliable and less computational intensive with high accuracy.
- The EHDS avoids overfitting as it is validated through 5-, 10-, 15-, and 20-fold cross-validation.
- The proposed approach does not only rely on morphological characteristics of the acquired signal.
- The method can be completely automated, and it works with all qualities of PuPG signals.

Despite the enormous advantages of the proposed method, it has a few limitations.

- The data set used in this research is yet small, with each sample with a length of 10 s.
- The procedure of initial feature extraction and selection of proper IMFs in EMD made the overall process strenuous and time-consuming.

The proposed study conducted a comprehensive comparison of preprocessing schemes (DWT and EMD), feature analysis, selection, and classification as illustrated in Figure 3. The computational complexity of the proposed is significantly low due to the fact that it operates on trained classifier models, therefore eliminating the training computational cost (Figure 19). The proposed system has the potential to be deployed in clinical environments and intensive care units where it can contribute to lessen the workload of medical professionals through its accurate detection and timely diagnosis. In future works, our research group aims to increase the data set size and apply deep learning models to automate the feature extraction process. The proposed framework is intended to be implemented on portable embedded platforms.

## Figures and Tables

**Table 1:** Categorization of blood pressure.

Class	Systolic (mmHg)	Diastolic (mmHg)
Optimal	Less than 120	Less than 80
Normal	120 to 129	80 to 84
High Normal	130 to 139	85 to 89
Hypertension	More than or equal to 140	More than or equal to 90

**Table 2:** Difference between PPG and PuPG data acquisition.

Type	Photoplethysmograph (PPG) Sensor	Pulse Plethysmograph (PuPG) Sensor
Input signal	Optical signal	Pressure changes
Phenomenon	Blood volumetric changes are detected by measuring the amount of light transmitted or reflected by the sensor.	Blood volumetric changes are detected by the piezoelectric material of the sensor as pressure changes when the blood volume changes.
Noise Impact	Light signal can be easily impacted by any external light changes. Dirty hand can distort the light intensities.	Piezoelectric material based sensors are normaly temperature sensitive. Dirty hands or foreign material on hand or fingers does not have significant impact.

**Table 3:** Summary of the self-collected PuPG data set.

Data Class	Subjects	Age Group	Samples
Hypertension	Male: 29	Male: 40–76	700
	Female: 27	Female: 39–59	
Normal	Male: 35	Male: 21–63	709
	Female: 30	Female: 20–59	
Overall	121	20–76	1409

**Table 4:** Comparison of mean relative energies and frequency ranges of various decomposition levels for Normal and Hypertension classes.

Decomposition Levels	Frequency Range (Hz)	Mean Relative Energy (%)	
		Normal	Hypertension
$D_{1}$	250–500	0.07%	0.59%
$D_{2}$	122–256	0.09%	0.32%
$D_{3}$	61.1–128	0.19%	0.35%
$D_{4}$	30.6–63.9	0.46%	0.49%
$D_{5}$	15.3–31.9	1.93%	3.10%
$D_{6}$	7.65–16	14.77%	13.31%
$D_{7}$	3.84–7.97	31.03%	21.49%
$D_{8}$	1.94–3.99	29.11%	19.05%
$D_{9}$	1.03–1.99	21.11%	26.77%
$D_{10}$	0.594–0.958	0.16%	7.65%
$A_{10}$	0–0.431	1.08%	6.88%
$A_{4}$	0–31.2	99.19%	98.26%

**Table 5:** Comparison of mean relative energies and frequency ranges of various intrinsic mode functions (EMD) for Normal and Hypertension classes. Bold font indicates the selected components.

Components	Normal		Hypertension	
	Mean Frequency Range (Hz)	Mean Relative Energy (%)	Mean Frequency Range (Hz)	Mean Relative Energy (%)
$IMF_{1}$	103–483	0.00	86.5–484	1.02
**$IMF_{2}$**	**11.3–60.2**	**0.14**	**40.7–219**	**0.35**
**$IMF_{3}$**	**3.09–14**	**30.34**	**3.3–61**	**2.04**
**$IMF_{4}$**	**2.99–12.2**	**4.97**	**3.34–23.3**	**6.65**
**$IMF_{5}$**	**1.28–10**	**22.76**	**2.98–11.4**	**14.95**
**Residual**	**0.129–5.55**	**41.78**	**0.0197–4.33**	**74.99**

**Table 6:** Statistical data of all extracted features for both methods.

	Method I				Method II			
**Feature**	**Normal**		**Hypertension**		**Normal**		**Hypertension**	
	**Mean**	**STD**	**Mean**	**STD**	**Mean**	**STD**	**Mean**	**STD**
Mean	0.008	0.027	0.017	0.031	0.001	0.049	0.013	0.053
Standard Deviation	0.253	0.032	0.250	0.046	0.254	0.032	0.248	0.045
Skewness	−1.959	0.522	−2.144	0.651	−1.997	0.576	−2.220	0.641
Kurtosis	6.993	1.499	8.083	3.864	7.139	1.688	8.297	3.944
Peak to Peak Value	1.380	0.199	1.398	0.140	1.377	0.178	1.375	0.144
Root Mean Square	0.255	0.033	0.252	0.047	0.258	0.038	0.254	0.046
Crest Factor	1.659	0.939	1.635	0.381	1.587	0.991	1.523	0.536
Shape Factor	1.484	0.134	1.458	0.159	1.522	0.163	1.465	0.167
Impulse Factor	2.435	1.297	2.382	0.593	2.371	1.376	2.171	0.632
Margin Factor	15.22	14.64	15.12	7.27	15.16	15.56	13.39	6.45
Energy	389.6	209.7	437.4	207.6	393.9	207.4	448.5	222.5
Peak to RMS Value	3.894	0.623	4.094	0.991	3.921	0.663	4.108	1.102
Root Sum of Squares	18.933	5.600	20.292	5.089	19.069	5.525	20.480	5.412
Shannon Energy	549.7	312.3	618.2	279.7	526.6	295.8	686.5	414.4
Log Energy	−27,888	15,515	−34,569	18,644	−28,509	16,289	−32,613	16,387
Mean Absolute Deviation	0.169	0.026	0.169	0.043	0.169	0.028	0.168	0.043
Median Absolute Deviation	0.074	0.026	0.071	0.030	0.071	0.033	0.059	0.024
Average Frequency	0.002	0.001	0.001	0.001	0.002	0.003	0.002	0.002
Jitter	137.1	180.9	85.5	150.4	159.0	248.8	52.0	95.8
Spectral Mean	3.144	5.928	0.771	1.960	8.623	15.700	2.764	6.372
Spectral Standard Deviation	3.401	4.833	1.549	3.463	7.571	11.498	3.031	6.016
Spectral Skewness	2.361	1.496	3.763	1.295	0.996	1.360	1.797	1.908
Specral Kurtosis	11.331	8.235	20.190	9.589	5.588	4.947	10.564	8.751
Spectral Centroid	9.442	0.202	9.915	1.481	9.771	1.576	10.768	2.216
Spectral Flux	0.008	0.002	0.008	0.002	0.008	0.002	0.008	0.002
Spectral Roll-off	91.699	1.010	96.587	8.383	97.036	10.371	136.257	44.372
Spectral Flatness	0.025	0.014	0.051	0.035	0.063	0.065	0.171	0.138
Spectral Crest	0.642	0.012	0.621	0.040	0.631	0.030	0.593	0.059
Spectral Decrease	−4.333	0.228	−4.013	0.658	−4.169	0.538	−3.656	0.830
Spectral Slope	−0.023	0.003	−0.023	0.003	−0.024	0.003	−0.023	0.005
Spectral Spread	18.505	0.199	19.029	1.178	19.328	1.617	23.521	5.818
Mean Frequency	4.347	0.949	4.999	3.451	4.989	3.063	6.446	4.440
Median Frequency	3.574	0.803	2.972	0.880	3.811	3.573	2.846	0.753
Spurious-free Dynamic Range	3.073	6.019	2.196	2.107	3.163	6.451	2.056	1.812
Signal to Noise Distortion	−0.885	6.041	−2.036	3.101	−0.755	6.819	−2.097	3.030
Total Harmonic Distortions	−2.376	5.452	−0.938	4.964	−3.144	6.562	−0.396	4.110
1st Coeffient of MFCC	−44.99	0.37	−44.716	0.512	−44.84	0.60	−44.45	0.80
2nd Coeffient of MFCC	6.268	0.523	6.661	0.714	6.480	0.846	7.028	1.122
3rd Coeffient of MFCC	5.976	0.499	6.350	0.683	6.169	0.802	6.690	1.066
4th Coeffient of MFCC	5.508	0.462	5.851	0.634	5.671	0.733	6.148	0.976
1st Coeffient of GFCC	−7.183	0.430	−6.762	0.610	−7.027	0.793	−6.220	1.266
2nd Coeffient of GFCC	1.844	0.063	1.869	0.071	1.522	0.419	1.119	0.574
3rd Coeffient of GFCC	0.553	0.138	0.367	0.269	0.643	0.105	0.492	0.206
4th Coeffient of GFCC	0.301	0.024	0.266	0.033	0.392	0.109	0.408	0.109
1st Coefficient of Chroma Vector	0.383	0.235	0.750	0.501	0.653	0.532	2.126	1.930
2nd Coefficient of Chroma Vector	0.416	0.258	0.773	0.518	0.663	0.546	2.130	1.948
3rd Coefficient of Chroma Vector	0.433	0.269	0.842	0.575	0.742	0.672	2.129	2.144
4th Coefficient of Chroma Vector	0.623	0.378	1.297	0.942	0.700	0.568	2.011	2.046
5th Coefficient of Chroma Vector	0.564	0.337	1.212	0.872	0.691	0.534	2.044	1.935
6th Coefficient of Chroma Vector	0.527	0.320	1.230	0.987	0.748	0.563	2.227	2.267
7th Coefficient of Chroma Vector	0.483	0.296	1.069	0.760	0.705	0.524	2.107	1.893
8th Coefficient of Chroma Vector	0.451	0.279	0.982	0.696	0.686	0.528	2.071	1.863
9th Coefficient of Chroma Vector	0.429	0.268	0.908	0.638	0.679	0.529	2.099	1.929
10th Coefficient of Chroma Vector	0.400	0.251	0.878	0.609	0.651	0.537	2.087	1.848
11th Coefficient of Chroma Vector	0.373	0.232	0.776	0.537	0.668	0.522	2.106	1.954
12th Coefficient of Chroma Vector	0.348	0.225	0.705	0.474	0.622	0.522	2.078	1.890
Enhanced Mean Absolute Value	0.297	0.039	0.302	0.055	0.294	0.052	0.301	0.049
Enhanced Wavelength	236.4	133.5	413.7	319.8	284.3	198.6	665.7	515.0
Wavelength	36.83	21.94	85.59	96.94	54.42	46.94	193.47	186.80
Slope Sign Change	45.1	86.9	508.4	549.0	1039.5	1657.6	3463.1	2792.1
Average Amplitude Change	0.006	0.003	0.010	0.009	0.009	0.009	0.021	0.018
Difference Absolute Std. Dev.	0.009	0.003	0.013	0.010	0.014	0.010	0.027	0.021
Log Detector	0.108	0.030	0.118	0.037	0.107	0.043	0.117	0.036
Modified Mean Absolute Value	0.130	0.025	0.133	0.034	0.130	0.033	0.132	0.030
Modified Mean Absolute Value 2	0.083	0.022	0.089	0.026	0.084	0.027	0.087	0.020
Pulse Percentage Rate	0.939	0.029	0.953	0.027	0.937	0.045	0.957	0.035
Simple Square Integral	389.6	209.7	437.4	207.6	393.9	207.4	448.5	222.5
Willison Amplitude	1153.6	765.6	2670.8	2603.9	1836.0	1707.6	4551.3	3502.4
Maximum Fractal Length	−0.463	0.384	−0.136	0.740	−0.174	0.594	0.428	1.121
Root Squared Zero Order Moment	2.592	0.032	2.600	0.026	2.593	0.031	2.601	0.028
Root Squared 2nd Order Moment	2.068	0.066	2.036	0.062	1.984	0.115	1.913	0.130
Root Squared 4th Order Moment	2.045	0.077	2.001	0.078	1.891	0.159	1.794	0.205
Sparseness	0.535	0.064	0.582	0.086	0.655	0.137	0.747	0.188
Irregularity Factor	−0.464	0.037	−0.445	0.047	−0.446	0.058	−0.406	0.061
Waveform Length Ratio	−0.065	0.703	−0.354	0.230	−0.648	0.604	−0.721	0.727
Complexity	0.502	0.222	0.706	0.253	0.897	0.524	1.314	0.515
Mobility	0.038	0.011	0.057	0.038	0.055	0.035	0.115	0.079
Higuchi’s Fractal Dimension	1.054	0.052	1.149	0.119	1.183	0.240	1.490	0.401
Katz Fractal Dimension	1.000	0.000	1.000	0.000	1.000	0.000	1.000	0.000
Lyapunov Exponent	437.4	49.5	394.8	49.3	362.9	78.8	239.1	92.2
Approximate Entropy	0.104	0.049	0.155	0.134	0.127	0.080	0.316	0.312
Correlation Dimension	1.687	0.168	1.733	0.191	1.676	0.202	1.731	0.300
1st Coefficient of LTP	259.6	128.8	291.5	122.1	259.6	128.8	291.5	122.1
2nd Coefficient of LTP	40.790	30.943	56.809	35.998	40.790	30.943	56.809	35.998
3rd Coefficient of LTP	24.574	23.717	41.723	32.083	24.574	23.717	41.723	32.083
4th Coefficient of LTP	16.381	15.761	30.738	22.667	16.381	15.761	30.738	22.667
5th Coefficient of LTP	18.472	15.755	33.411	24.881	18.472	15.755	33.411	24.881
6th Coefficient of LTP	25.205	21.607	45.518	33.715	25.205	21.607	45.518	33.715
7th Coefficient of LTP	17.699	16.352	29.035	22.609	17.699	16.352	29.035	22.609
8th Coefficient of LTP	26.261	23.075	39.645	28.386	26.261	23.075	39.645	28.386
9th Coefficient of LTP	47.483	32.844	58.163	35.132	47.483	32.844	58.163	35.132
10th Coefficient of LTP	207.278	94.769	201.809	72.537	207.278	94.769	201.809	72.537
11th Coefficient of LTP	269.5	137.7	302.1	121.7	269.5	137.70	302.1	121.7
12th Coefficient of LTP	41.733	31.354	59.773	38.092	41.733	31.354	59.773	38.092
13th Coefficient of LTP	24.006	23.096	41.071	31.294	24.006	23.096	41.071	31.294
14th Coefficient of LTP	16.784	15.900	30.199	23.471	16.784	15.900	30.199	23.471
15th Coefficient of LTP	18.506	15.140	33.390	25.583	18.506	15.140	33.390	25.583
16th Coefficient of LTP	26.148	22.755	44.177	31.682	26.148	22.755	44.177	31.682
17th Coefficient of LTP	16.898	16.559	30.149	21.910	16.898	16.559	30.149	21.910
18th Coefficient of LTP	25.790	22.892	39.312	26.784	25.790	22.892	39.312	26.784
19th Coefficient of LTP	46.534	33.045	56.298	33.647	46.534	33.045	56.298	33.647
20th Coefficient of LTP	197.773	85.090	191.858	74.487	197.773	85.090	191.858	74.487

**Table 7:** List of features extracted for method I and sorted with respect to mean rank (MR) value. Bold font indicates the top 24 ranked features.

Feature	TT	KLD	BD	ROC	MWT	MRMR	RRF	MR
**3rd Coefficient of LTP**	12	93	93	83	32	102	98	73.29
**6th Coefficient of Chroma Vector**	38	98	76	79	78	94	49	73.14
**Lyapunov Exponent**	93	92	92	70	81	10	70	72.57
**Sparseness**	70	90	90	30	62	79	84	72.14
**Jitter**	78	58	58	74	77	85	74	72.00
**9th Coefficient of LTP**	57	83	83	75	82	92	24	70.86
**Spectral Decrease**	62	28	61	85	75	93	83	69.57
**4th Coefficient of MFCC**	96	41	41	95	40	72	99	69.14
**Irregularity**	35	94	100	57	88	90	13	68.14
**1st Coeffient of MFCC**	99	23	23	100	37	96	97	67.86
**Waveform Length Ratio**	13	100	94	91	9	100	66	67.57
**3rd Coeffient of MFCC**	79	97	97	84	39	55	17	66.86
**1st Coefficient of Chroma Vector**	71	86	86	98	26	48	53	66.86
**Spectral Roll-off**	81	43	79	66	56	59	81	66.43
**Spectral Crest**	61	80	60	26	92	71	75	66.43
**6th Coefficient of Chroma Vector**	100	76	99	31	61	45	52	66.29
**7th Coefficient of Chroma Vector**	37	99	98	1	96	42	86	65.57
**Median Frequency**	88	35	78	71	102	78	2	64.86
**2nd Coefficient of Chroma Vector**	89	7	96	89	41	81	51	64.86
**Spectral Centroid**	76	79	43	39	86	69	58	64.29
**Difference Absolute Std. Dev. Value**	84	38	37	81	79	63	64	63.71
**Shape Factor**	59	53	53	54	72	80	73	63.43
**Spectral Mean**	43	77	77	96	46	40	61	62.86
**Simple Square Integral**	4	72	72	22	89	89	92	62.86
3rd Coefficient of GFCC	95	29	88	94	34	53	45	62.57
4th Coefficient of Chroma Vector	40	96	30	87	85	52	47	62.43
Root Mean Square	45	56	56	77	43	87	69	61.86
Signal to Noise Distortion	97	69	69	47	74	31	42	61.29
9th Coefficient of Chroma Vector	72	12	95	88	27	76	57	61.00
Mean Absolute Deviation	58	48	48	49	76	82	62	60.43
Root Squared 2nd Order Moment	91	82	71	8	59	20	91	60.29
Root Squared 4th Order Moment	101	71	82	82	4	66	14	60.00
10th Coefficient of Chroma Vector	36	95	85	28	73	41	56	59.14
12th Coefficient of Chroma Vector	90	89	89	59	10	37	38	58.86
1st Coefficient of GFCC	69	87	87	61	66	38	3	58.71
Mean	80	26	26	80	80	68	44	57.71
Enhanced Mean Absolute Value	21	73	73	62	58	54	63	57.71
Root Sum of Squares	53	49	52	99	18	58	72	57.29
2nd Coefficient of LTP	82	70	70	35	36	12	95	57.14
Katz Fractal Dimension	67	3	3	67	90	75	90	56.43

**Table 8:** List of features extracted for method II and sorted with respect to MR value. Bold font indicates the top 24 ranked features.

Feature	TT	KLD	BD	ROC	MWT	MRMR	RRF	MR
**7th Coefficient of Chroma Vector**	100	98	98	26	96	97	55	81.43
**4th Coefficient of Chroma Vector**	89	96	76	73	85	52	96	81.00
**Mobility**	93	64	64	67	68	91	95	77.43
**Spectral Centroid**	68	78	78	77	45	98	88	76.00
**Enhanced Mean Absolute Value**	71	95	95	28	98	56	87	75.71
**9th Coefficient of LTP**	64	101	101	64	93	79	19	74.43
**1st Coefficient of GFCC**	95	35	97	98	38	99	54	73.71
**7th Coefficient of LTP**	57	93	93	57	101	24	89	73.43
**Slope Sign Change**	74	73	89	79	89	89	13	72.29
**Maximum Fractal Length**	3	72	74	91	90	90	70	70.00
**3rd Coefficient of MFCC**	38	97	22	95	102	80	53	69.57
**6th Coefficient of Chroma Vector**	69	80	96	90	27	68	56	69.43
**8th Coefficient of Chroma Vector**	61	99	99	69	66	49	40	69.00
**Enhanced Wavelength**	81	85	85	68	94	16	50	68.43
**Pulse Percentage Rate**	4	94	100	42	73	76	77	66.57
**Root Squared Zero Order Moment**	63	84	84	30	69	72	46	64.00
**Crest Factor**	51	68	68	80	72	32	72	63.29
**Modified Mean Absolute Value 2**	42	90	90	7	88	64	62	63.29
**Spectral Crest**	87	58	45	40	47	85	75	62.43
**2nd Coeffient of MFCC**	37	69	69	84	77	48	51	62.14
**1st Coeffient of MFCC**	77	9	9	100	99	38	102	62.00
**Average Frequency**	80	29	54	47	54	86	82	61.71
**4th Coefficient of LTP**	34	65	65	65	42	74	84	61.29
**Willison Amplitude**	91	74	72	15	16	77	83	61.14
Spectral Spread	99	47	58	85	60	9	68	60.86
3rd Coefficient of LTP	65	91	91	5	65	2	101	60.00
Root Squared 4th Order Moment	70	66	71	14	97	62	36	59.43
Lyapunov Exponent	32	63	63	8	62	81	100	58.43
2nd Coeffient of GFCC	40	87	87	89	40	50	14	58.14
3rd Coeffient of GFCC	73	22	10	66	39	96	99	57.86
Correlation Dimension	14	70	70	101	57	1	91	57.71
Root Squared 2nd Order Moment	101	5	66	36	31	100	61	57.14
5th Coefficient of Chroma Vector	20	76	80	97	4	36	86	57.00
2nd Coefficient of Chroma Vector	75	41	41	88	78	73	2	56.86
11th Coefficient of Chroma Vector	66	39	39	74	95	47	37	56.71
Log Energy	45	75	52	53	19	88	60	56.00
10th Coefficient of Chroma Vector	90	38	38	19	100	69	38	56.00
5th Coefficient of LTP	8	82	82	83	5	101	30	55.86
1st Coefficient of LTP	11	92	92	24	83	57	31	55.71

**Table 9:** Consolidated result analysis of feature sets ($S_{1}$, $S_{2}$, $S_{3}$) for method I with various classifiers.

Classifier	$S_{1}$ (5 Components)				$S_{2}$ (7 Components)				$S_{3}$ (10 Components)			
	Acc	Sp	Sen	Err	Acc	Sp	Sen	Err	Acc	Sp	Sen	Err
DT	0.874	0.89	0.89	0.126	0.924	0.92	0.93	0.076	0.934	0.94	0.93	0.066
LD	0.688	0.3	1	0.312	0.688	0.3	1	0.312	0.669	0.3	0.97	0.331
LR	0.688	0.3	1	0.312	0.688	0.3	1	0.312	0.688	0.3	1	0.312
NBG	0.688	0.3	1	0.312	0.688	0.3	1	0.312	0.59	1	0.26	0.41
NBK	0.804	0.91	0.72	0.196	0.83	0.92	0.76	0.17	0.893	0.91	0.88	0.107
SVM-L	0.479	0.56	0.41	0.521	0.587	0.11	0.97	0.413	0.498	0.37	0.6	0.502
SVM-Q	0.527	0.65	0.43	0.473	0.527	0.08	0.89	0.473	0.546	0.41	0.65	0.454
SVM-C	0.47	0.4	0.53	0.53	0.555	0.03	0.98	0.445	0.524	0	0.94	0.476
SVM-FG	0.688	0.3	1	0.312	0.688	0.30	1	0.312	0.688	0.3	1	0.312
SVM-MG	0.688	0.3	1	0.312	0.688	0.30	1	0.312	0.688	0.3	1	0.312
KNN-F	0.937	0.9	0.97	0.063	0.972	0.96	0.98	0.028	0.984	0.97	0.99	0.016
KNN-M	0.792	0.68	0.88	0.208	0.864	0.81	0.91	0.136	0.905	0.86	0.94	0.095
KNN-Cos	0.685	0.3	0.99	0.315	0.681	0.3	0.99	0.319	0.685	0.3	0.99	0.315
KNN-C	0.672	0.68	0.66	0.328	0.871	0.83	0.9	0.129	0.896	0.84	0.94	0.104
KNN-W	0.921	0.88	0.95	0.079	0.965	0.96	0.97	0.035	0.978	0.97	0.98	0.022
Eboost	0.918	0.89	0.94	0.082	0.864	0.74	0.96	0.136	0.555	0	1	0.445
EBT	0.688	0.3	1	0.312	0.972	0.95	0.99	0.028	0.943	0.93	0.95	0.057
ESD	0.94	0.92	0.95	0.06	0.688	0.3	1	0.312	0.681	0.3	0.99	0.319
ESKNN	0.915	0.91	0.91	0.085	0.984	0.98	0.99	0.016	0.981	0.97	0.99	0.019

**Table 10:** Consolidated result analysis of feature sets ($S_{4}$, $S_{5}$, $S_{6}$) for method I with various classifiers. Bold font indicates best results.

Classifier	$S_{4}$ (12 Components)				$S_{5}$ (15 Components)				$S_{6}$ (17 Components)			
	Acc	Sp	Sen	Err	Acc	Sp	Sen	Err	Acc	Sp	Sen	Err
DT	0.959	0.96	0.96	0.041	0.959	0.94	0.98	0.041	0.972	0.96	98	0.028
LD	0.581	0.3	0.99	0.419	0.662	0.3	0.95	0.338	0.691	0.32	0.99	0.309
LR	0.688	0.3	1	0.312	0.688	0.3	1	0.312	0.675	0.35	0.94	0.325
NBG	0.59	1	0.26	0.41	0.625	1	0.32	0.375	0.631	1	0.34	0.369
NBK	0.868	0.85	0.89	0.132	0.877	0.92	0.84	0.123	0.88	0.91	0.95	0.12
SVM-L	0.524	0.26	0.74	0.476	0.543	0.12	0.88	0.457	0.536	0.07	0.91	0.464
SVM-Q	0.552	0.22	0.82	0.448	0.536	0.33	0.7	0.464	0.546	0.02	0.97	0.454
SVM-C	0.524	0.8	0.88	0.476	0.517	0	0.93	0.483	0.514	0.05	0.89	0.486
SVM-FG	0.688	0.3	1	0.312	0.688	0.3	1	0.312	0.7	0.33	1	0.3
SVM-MG	0.665	0.3	1	0.335	0.688	0.3	1	0.312	0.694	0.31	1	0.306
KNN-F	0.981	0.97	0.99	0.019	0.975	0.97	0.98	0.025	0.915	0.88	0.94	0.085
KNN-M	0.918	0.85	0.97	0.082	0.912	0.85	0.96	0.088	0.659	0.7	0.63	0.341
KNN-Cos	0.688	0.3	1	0.312	0.688	0.3	1	0.312	0.685	0.3	0.99	0.315
KNN-C	0.905	0.85	0.95	0.095	0.909	0.85	0.95	0.091	0.909	0.89	0.93	0.091
KNN-W	0.981	0.98	0.98	0.019	0.975	0.97	0.98	0.025	0.978	0.97	0.98	0.022
Eboost	0.555	0	1	0.445	0.555	0	1	0.445	0.555	0	1	0.445
EBT	0.965	0.94	0.98	0.035	0.972	0.97	0.97	0.028	0.94	0.92	0.95	0.06
ESD	0.688	0.3	1	0.312	0.666	0.3	0.96	0.334	0.681	0.3	0.99	0.319
**ESKNN**	**0.984**	**0.97**	**0.99**	**0.016**	0.975	0.97	0.98	0.025	0.981	0.99	0.99	0.019

**Table 11:** Validation of the selected scheme of method I.

Evaluation	Classes	Accuracy	True Positive Rate	False Negative Rate
5-Fold Cross-Validation	Healthy	0.983	0.98	0.02
	Hypertension		0.99	0.01
10-Fold Cross-Validation	Healthy	0.984	0.98	0.02
	Hypertension		0.99	0.01
15-Fold Cross-Validation	Healthy	0.984	0.98	0.02
	Hypertension		0.99	0.01
20-Fold Cross-Validation	Healthy	0.984	0.98	0.02
	Hypertension		0.99	0.01
20% Hold Out Validation	Healthy	0.978	1	0
	Hypertension		0.94	0.06
25% Hold Out Validation	Healthy	0.989	0.98	0.02
	Hypertension		1	0

**Table 12:** Feature analysis table ($S_{1}$, $S_{2}$, $S_{3}$) for method II. Bold font indicates the best results.

Classifier	$S_{1}$ (5 Components)				$S_{2}$ (7 Components)				$S_{3}$ (10 Components)			
	Acc	Sp	Sen	Err	Acc	Sp	Sen	Err	Acc	Sp	Sen	Err
DT	0.974	0.98	0.97	0.026	0.983	0.98	0.99	0.017	0.989	0.98	0.99	0.011
LD	0.619	0.24	1	0.381	0.619	0.24	1	0.381	0.568	0.24	0.9	0.432
LR	0.679	0.97	0.39	0.321	0.679	0.97	0.39	0.321	0.679	0.97	0.39	0.321
NBG	0.619	0.24	1	0.381	0.619	0.24	1	0.381	0.268	1	0.26	0.732
NBK	0.946	0.97	0.92	0.054	0.946	0.98	0.91	0.054	0.94	0.97	0.91	0.06
SVM-L	0.48	0.49	0.47	0.52	0.497	0.59	0.41	0.503	0.523	0.52	0.53	0.477
SVM-Q	0.51	0.53	0.5	0.49	0.511	0.24	0.78	0.489	0.497	0.46	0.53	0.503
SVM-C	0.49	0.3	0.69	0.51	0.491	0.22	0.77	0.509	0.491	0.23	0.76	0.509
SVM-FG	0.668	1	0.34	0.332	0.662	1	0.32	0.338	0.665	0.99	0.34	0.335
SVM-MG	0.619	0.24	1	0.381	0.614	0.51	0.72	0.386	0.597	0.73	0.46	0.403
KNN-F	0.99	0.99	0	0.01	0.893	0.98	0.984	0.107	0.991	0.99	0.99	0.009
KNN-M	0.957	0.93	0.98	0.043	0.969	0.95	0.99	0.031	0.972	0.95	0.99	0.028
KNN-Cos	0.631	0.27	0.99	0.369	0.639	0.3	0.98	0.361	0.636	0.3	0.98	0.364
KNN-C	0.957	0.93	0.98	0.043	0.966	0.94	0.99	0.034	0.969	0.94	0.99	0.031
**KNN-W**	**0.994**	**0.992**	**0.996**	**0.006**	0.986	0.94	0.99	0.014	0.992	0.99	0.99	0.008
Eboost	0.489	0.39	0.59	0.511	0.489	0.39	0.59	0.511	0.489	0.39	0.59	0.511
EBT	0.98	0.97	0.99	0.02	0.986	0.98	0.99	0.014	0.986	0.98	0.99	0.014
ESD	0.619	0.24	1	0.381	0.619	0.24	1	0.381	0.571	0.24	0.9	0.429
ESKNN	0.991	0.99	0.99	0.009	0.983	0.99	0.98	0.017	0.991	0.99	0.99	0.009

**Table 13:** Feature analysis table ($S_{4}$, $S_{5}$, $S_{6}$) for method II.

Classifier	$S_{4}$ (12 Components)				$S_{5}$ (15 Components)				$S_{6}$ (17 Components)			
	Acc	Sp	Sen	Err	Acc	Sp	Sen	Err	Acc	Sp	Sen	Err
DT	0.992	0.99	0.99	0.008	0.972	0.95	0.99	0.028	0.983	0.98	0.98	0.017
LD	0.548	0.32	0.78	0.452	0.565	0.34	0.8	0.435	0.665	1	0.33	0.335
LR	0.679	0.97	0.39	0.321	0.679	0.97	0.39	0.321	0.676	0.97	0.38	0.324
NBG	0.636	0.97	0.39	0.364	0.662	1	0.32	0.338	0.665	1	0.33	0.335
NBK	0.92	0.96	0.88	0.08	0.926	0.97	0.89	0.074	0.909	0.95	0.87	0.091
SVM-L	0.531	0.27	0.79	0.469	0.486	0.23	0.74	0.514	0.5	0.24	0.76	0.5
SVM-Q	0.503	0.19	0.82	0.497	0.469	0.15	0.79	0.531	0.514	0.15	0.88	0.486
SVM-C	0.472	0	0.94	0.528	0.472	0.1	0.85	0.528	0.486	0	0.97	0.514
SVM-F	0.662	1	0.32	0.338	0.662	1	0.32	0.338	0.696	1	0.39	0.304
SVM-MG	0.665	1	0.33	0.335	0.662	1	0.32	0.338	0.696	1	0.39	0.304
KNN-F	0.991	0.99	0.99	0.009	0.983	0.98	0.98	0.017	0.989	0.98	0.99	0.011
KNN-M	0.949	0.94	0.96	0.051	0.96	0.94	0.98	0.04	0.94	0.97	0.91	0.06
KNN-Cos	0.639	0.28	1	0.361	0.628	0.28	0.97	0.372	0.645	0.3	0.99	0.355
KNN-C	0.946	0.94	0.95	0.054	0.963	0.94	0.98	0.037	0.94	0.98	0.9	0.06
KNN-W	0.991	0.99	0.99	0.009	0.986	0.99	0.98	0.014	0.993	0.99	0.99	0.007
Eboost	0.489	0.39	0.59	0.511	0.534	0.48	0.59	0.466	0.489	0.39	0.59	0.511
EBT	0.989	0.98	0.99	0.011	0.966	0.97	0.96	0.034	0.986	0.99	0.98	0.014
ESD	0.577	0.26	0.89	0.423	0.563	0.39	0.73	0.437	0.665	1	0.33	0.335
ESKNN	0.991	0.99	0.99	0.009	0.983	0.98	0.98	0.017	0.991	0.99	0.99	0.009

**Table 14:** Validation of the selected scheme of method II.

Evaluation	Classes	Accuracy	True Positive Rate	False Negative Rate
5 Fold Cross-Validation	Healthy	0.986	0.99	0.01
	Hypertension		0.98	0.02
10 Fold Cross-Validation	Healthy	0.994	0.99	0.01
	Hypertension		>0.99	<0.01
15 Fold Cross-Validation	Healthy	0.994	0.99	0.01
	Hypertension		>0.99	<0.01
20 Fold Cross-Validation	Healthy	0.997	0.99	0.01
	Hypertension		1	0
20% Hold Out Validation	Healthy	0.986	1	0
	Hypertension		0.97	0.03
25% Hold Out Validation	Healthy	0.989	0.98	0.02
	Hypertension		1	0

**Table 15:** Performance comparison of methods I and II.

Performance	Method I	Method II
Accuracy	98.40%	99.40%
Sensitivity	97.00%	99.20%
Specificity	99.00%	99.60%
Error	0.02%	0.60%
# of features	12	5

**Table 16:** Comparison with previous works.

Ref.	Modality	Preprocessing	Features	Feature Reduction	Classification	Data Set	Results
[12]	PPG	CWT	GoogLeNet	-	GoogLeNet	MIMIC	F1 score: 92.55%
[13]	PPG and ECG	-	PAT and morphological features	-	KNN	MIMIC	F1 score: 94.84%
[14]	HRV	-	Standard deviation of NN intervals	-	MIL	Self-collected data set Hypertension 24 and Normal: 19	Accuracy: 85.47%
[15]	ECG	SGF	Entropy features	-	SVM	Self-collected data set Hypertension: 61 and Normal: 67	Accuracy: 93.33%
[16]	HRV	-	Statistical, spectral, geometrical, wavelet, fractal, and non-linear features	PCA	QDA	Self-collected data set Hypertension: 41 Normal: 30	Accuracy: 85.5%
[17]	ECG	OWFB	Fractal dimension and energy features	Student’s *t*-test	Diagnosis index	PhysioNet database High-risk Hypertension: 17 subjects Low-risk Hypertension: 122 subjects Total: 139 subjects	100% between low-risk and high-risk classes
[18]	ECG	EMD	Entropy features	Student’s *t*-test	KNN classifier	MIT BIH Sinus rhythm database, SHAREE database: Normal: 18 signals Hypertension: 139 signals	Accuracy: 97.70% Sensitivity: 98.90% Specificity: 89.10%
[19]	PPG	Chebyshev II	Time and morphological features	MRMR	KNN-W	Hypertension: 35 Normal: 48 Total: 83	Positive Predictive Value: 100% Sensitivity: 85.71% F1-score: 92.31%
[20]	BCG		Morphological features	-	CAR	Self-collected data set Hypertension: 61 and Normal: 67	Accuracy: 84.4%
This study	PuPG	EMD	Time, frequency, cepstral, fractal, and chaotic features	HFSR	KNN-W	Self-collected data set Hypertension: 56 Normal: 65	Accuracy: 99.7% Sensitivity: 99.2% Specificity: 99.4%

## Data Availability

Data will be available on request.
